# Reply: Deferred Strategy in SLE-APS After Successful Thrombolysis: A Safer First Step?

**DOI:** 10.1016/j.jaccas.2026.107046

**Published:** 2026-03-11

**Authors:** Rodrigo Uriel Palacios, Diego Costa, Maximiliano Muzzio, Roberto Coronel

We appreciate the thoughtful comments by Zhan^1^ regarding revascularization strategies in patients with systemic lupus erythematosus and suspected antiphospholipid syndrome (APS) presenting with ST-segment elevation myocardial infarction (STEMI). However, we respectfully disagree with the implication that immediate stent implantation was suboptimal in the reported case.

Primary percutaneous coronary intervention with stent implantation remains the guideline-recommended standard of care in STEMI, irrespective of underlying autoimmune disease, when a clear culprit lesion is identified. Achieving TIMI flow grade 3 after thrombolysis does not equate to plaque stabilization, nor does it exclude the presence of a ruptured, flow-limiting atherosclerotic lesion with a high risk of early reocclusion. In this context, mechanical stabilization of the culprit plaque by stenting directly addresses the pathophysiological substrate of infarction, rather than treating thrombus burden alone. DEFER-STEMI (Deferred Stenting in ST-Elevation Myocardial Infarction) enrolled highly selected patients with large thrombus burden and excluded complex coronary anatomy and high-risk lesions.^2^ Also, deferred stenting did not demonstrate a mortality benefit, and subsequent trials and meta-analyses have failed to support routine deferral as a default strategy.

Regarding APS being associated with increased thrombotic risk, this strengthens rather than weakens the rationale for definitive revascularization. Leaving a disrupted plaque untreated in a prothrombotic area may increase the likelihood of rethrombosis. Modern drug-eluting stents, combined with optimized antithrombotic regimens, have significantly reduced rates of stent thrombosis compared with earlier eras cited in historical APS series.

In acute STEMI, the priority remains rapid myocardial salvage; surgical planning can follow once hemodynamic stability and myocardial reperfusion are secured. Delaying revascularization in favor of immunosuppression and anticoagulation presupposes diagnostic certainty regarding APS reactivation and inflammatory activity, which cannot be definitively established in the hyperacute setting. In contrast, percutaneous coronary intervention offers immediate, reproducible, and evidence-based benefit, while allowing subsequent multidisciplinary optimization.

In summary, we contend that immediate stent implantation in this case was justified, guideline-concordant, and clinically appropriate; however, individualized strategies are essential in complex autoimmune patients.
